# Supplementary material for: Cardiomyocyte-Restricted Expression of IL11 Causes Cardiac Fibrosis, Inflammation, and Dysfunction
Source: Int J Mol Sci. 2023 Aug 20;24(16):12989. doi: 10.3390/ijms241612989 (PMC10455677; doi:10.3390/ijms241612989)
Supplement: Supplementary file 1 [file ijms-24-12989-s001.zip › ijms-2537279-supplementary.pdf]

Supplementary Figures

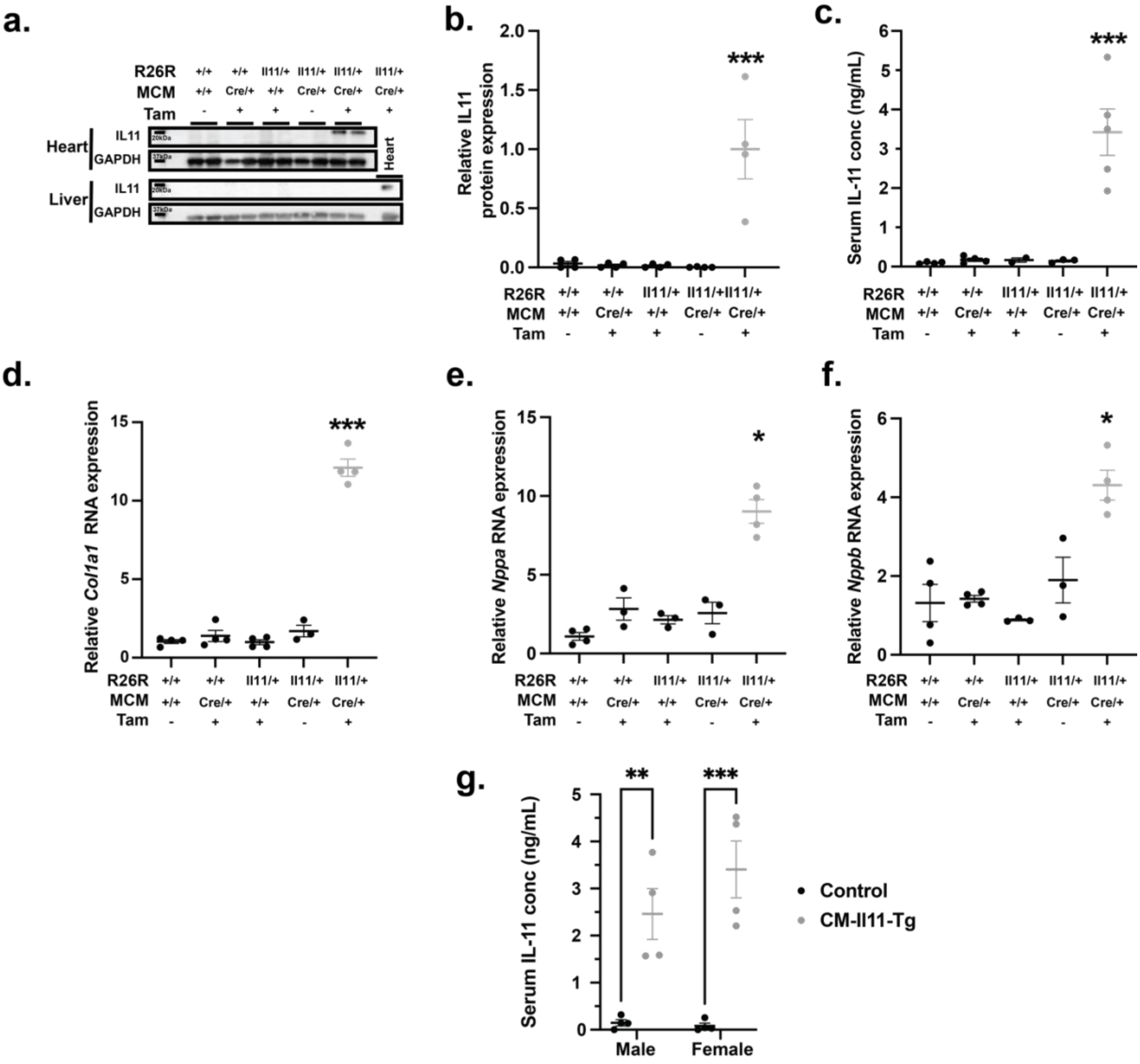

Supplementary Figure S1. (a) Western blot of IL11 expression in the ventricular myocardium and liver in male mice with different combinations of *R26R-Il11* transgene, MCM gene and tamoxifen administration. Demonstrating myocardial-specific expression in response to tamoxifen administration without detectable hepatic expression. **(b)** Quantification of western blot from myocardial western blot in (a) (n=4 per condition) **(c)** Serum IL11 protein concentration using colourimetric IL11 serum ELISA (n=3-4 per condition). QPCR of myocardial tissue from male mice targeting **(d)** *Col1a1,* **(e)** *Nppa and* **(f)** *Nppb* in combinations of genotypes and tamoxifen administration (n=3-4 per condition). **(g)** Serum IL11 protein concentration measured by ELISA of male and female, control (●) and CM-Il11-Tg (●) mice 6 weeks after tamoxifen administration (n=4 per group).

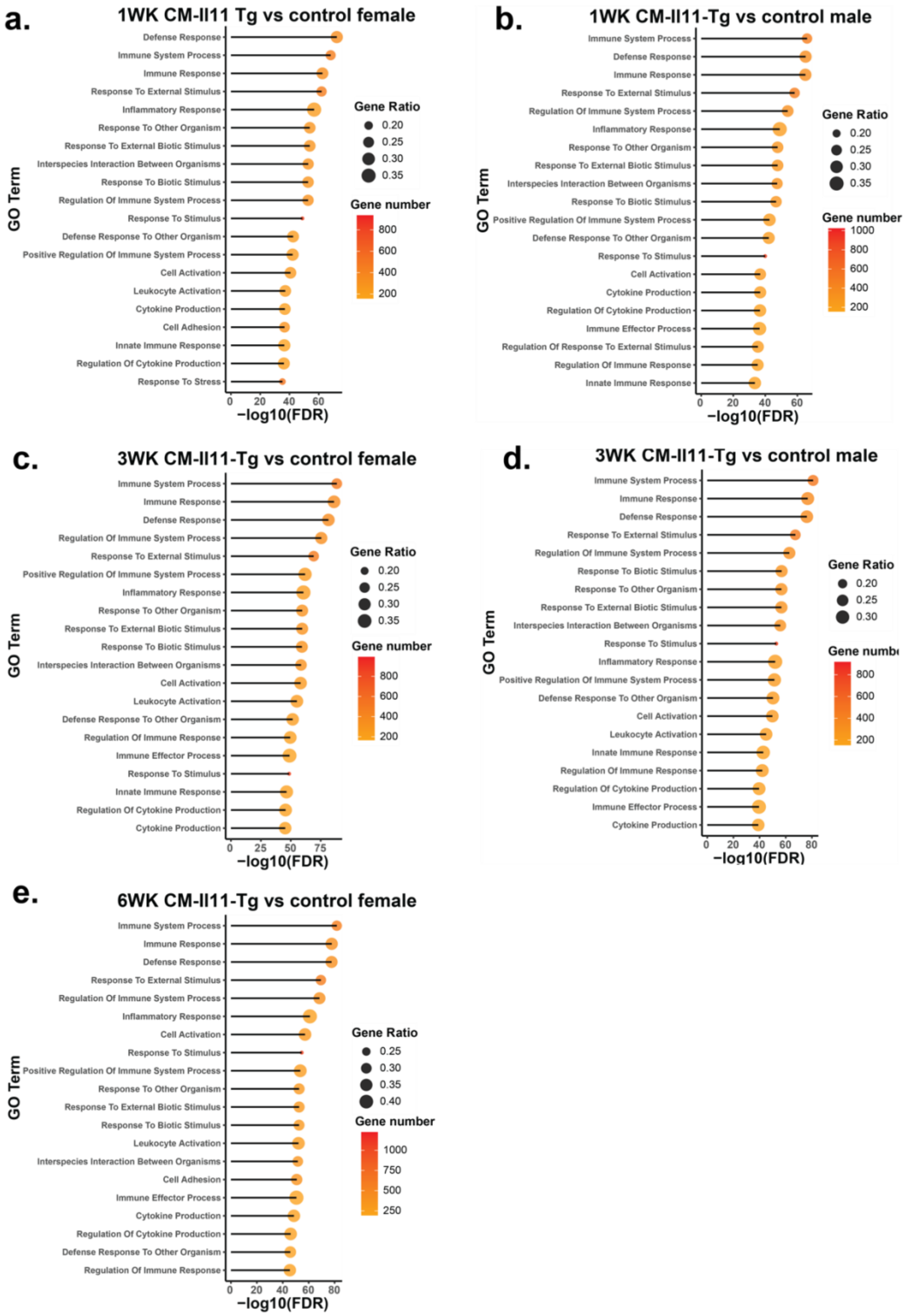

Supplementary Figure S2. Top 20 Biological processes GO terms. Top 20 gene ontology biological processes terms from gene ontology analysis of differentially expressed genes in male and female CM-Il11-Tg mice compared to controls at 1, 3, and 6 weeks after administration of tamoxifen.

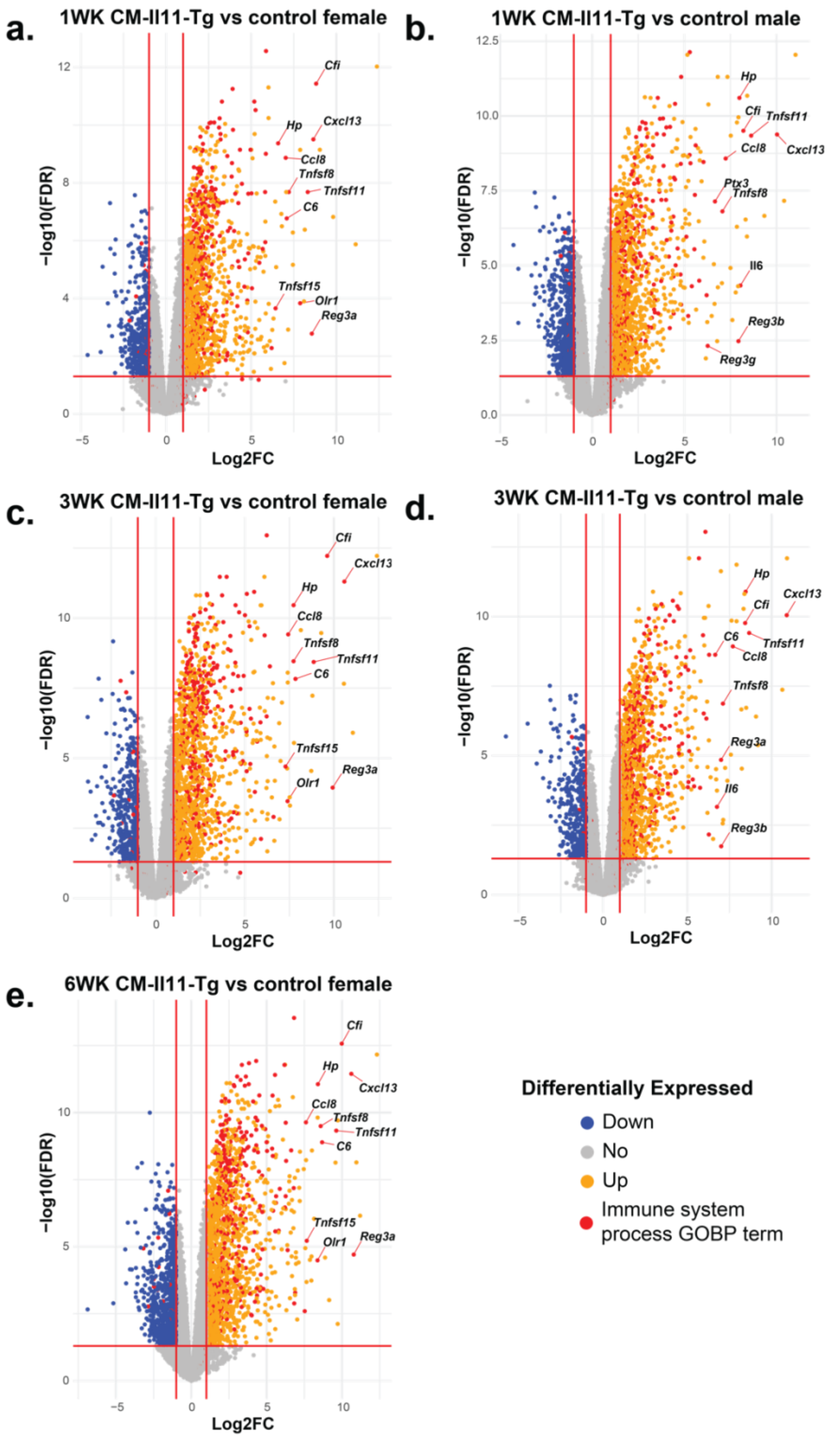

Supplementary Figure S3. Volcano plot of differentially expressed genes. Visualisation of all detected genes in myocardial RNA seq data in males and female CM-Il11-Tg mice compared to controls at 1, 3 and 6 weeks after administration of tamoxifen. Red lines are placed at log2Fc of 1 or -1 and at a false discovery rate of 0.05. Genes involved in the most enriched GOBP term "immune system processes" are highlighted in red and the top 10 genes in this GO term are labelled (n=4vs4 per time point).

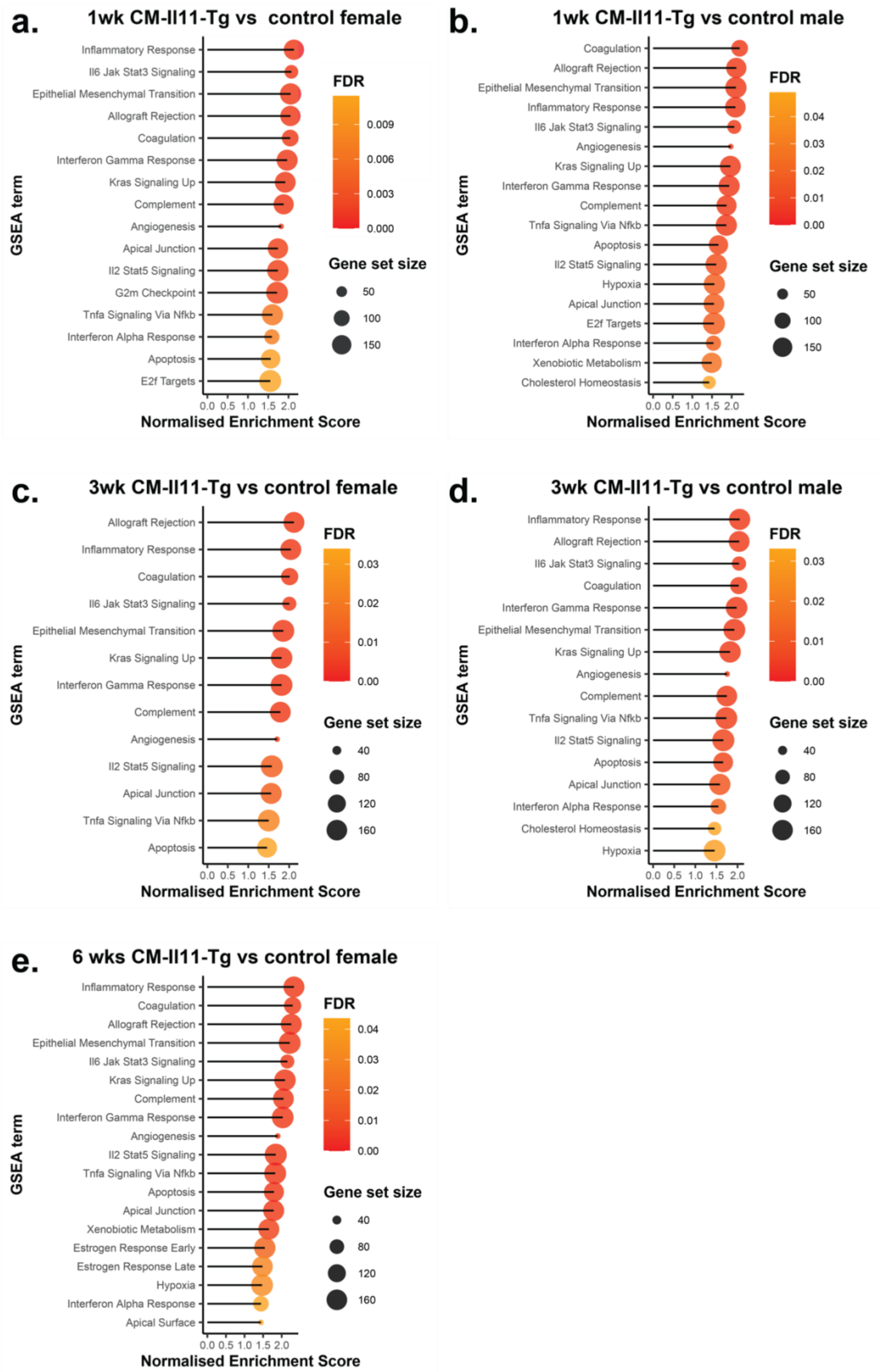

Supplementary Figure S4. Gene set enrichment analysis using Hallmark gene sets. Upregulated hallmark gene sets using GSEA in male and female CM-Il11-Tg mice compared to controls at 1, 3 and 6 weeks after tamoxifen administration.

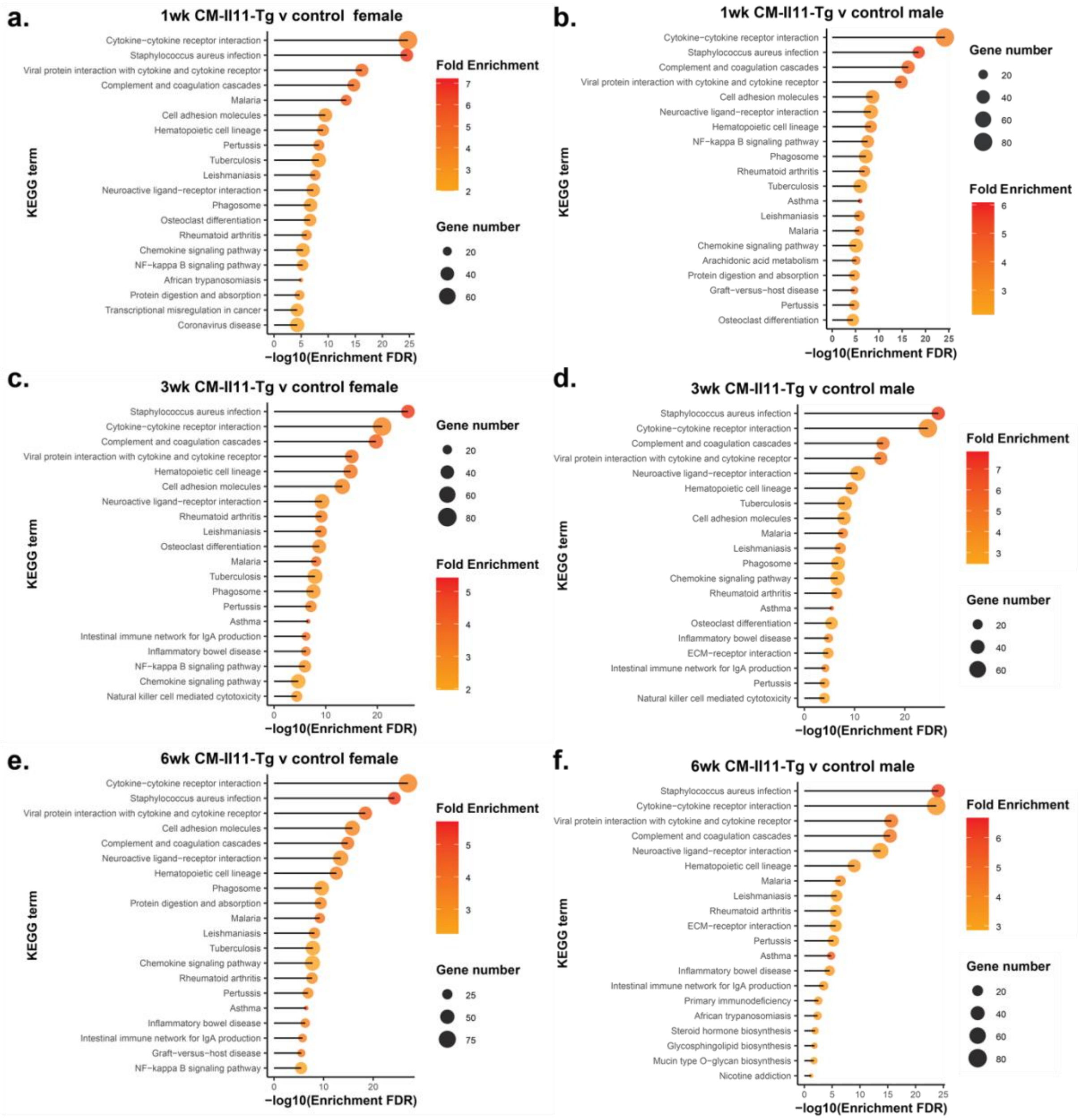

Supplementary Figure S5. KEGG analysis at individual time points. Top 20 most significantly enriched KEGG terms based on differentially expressed genes in male and female CM-Il11-Tg mice compared to controls at 1, 3, and 6 weeks after tamoxifen administration.

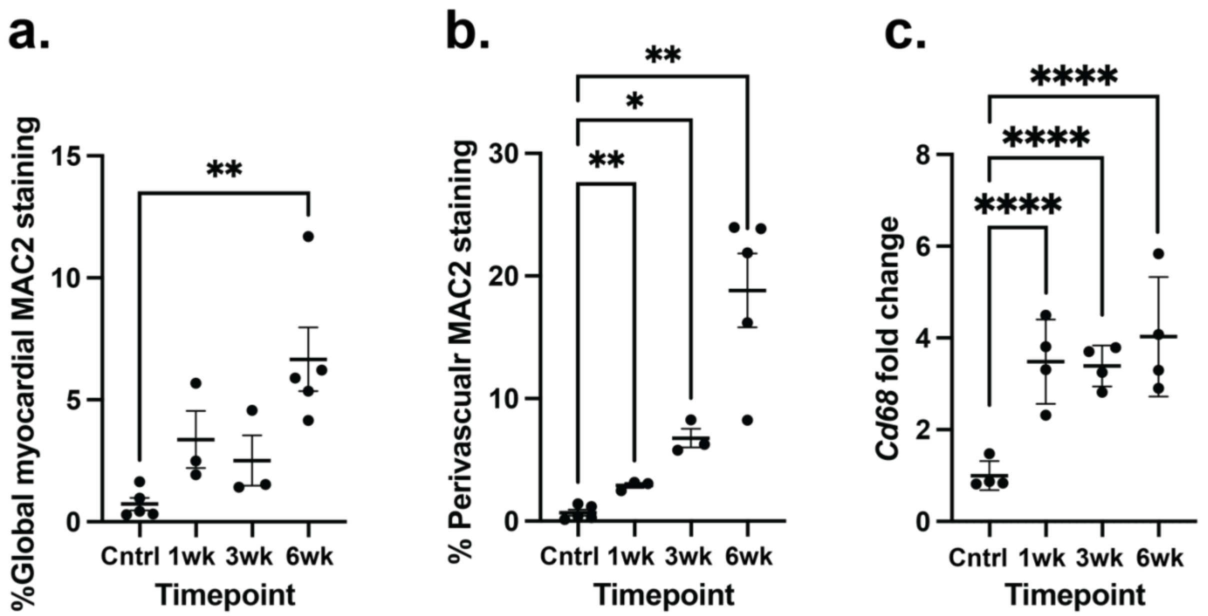

Supplementary Figure S6. Quantification of macrophage infiltration. (a) Quantification of MAC2 staining as a proportion of total myocardium in control (Cntrl) mice compared to CM-Il11-Tg mice 1, 3, and 6 weeks after induction of *Il11* expression (n=3-5 per timepoint) **(b)** Quantification of proportion of Mac2 staining in perivascular region of myocardial sections. (n=3-5 per timepoint) **(c)** Fold change of *Cd68* expression from RNA-seq data in WT and CM-Il11-Tg mice 1, 3, and 6 weeks after induction of *Il11* expression (n=4 per timepoint). Statistics: Comparison between WT and CM-Il11-Tg timepoints performed with one-way ANOVA with Sidak's multiple comparison tests. Differential gene expression was analysed using EdgeR software and corrected using false discovery rate method. Significance denoted as *p<0.05, **p<0.01, ***p<0.001.

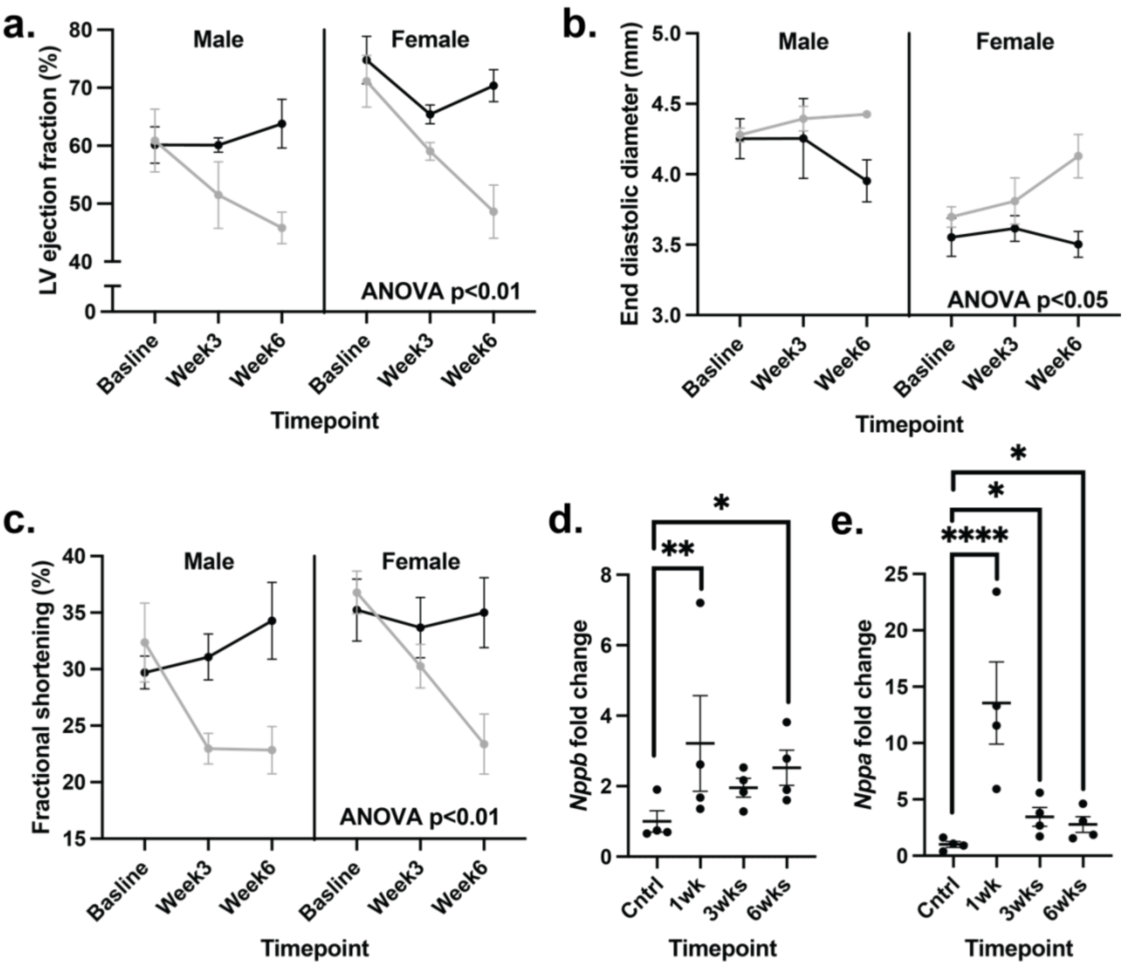

Supplementary Figure S7. Cardiac function changes compared to baseline at 3 weeks and 6 weeks. (a) Left ventricular ejection fraction in male and female control (●) and CM-Il11-Tg (●) mice at baseline, 3- and 6-weeks after transgene recombination. **(b)** End diastolic volume at baseline, 3- and 6-weeks. **(c)** Fractional shortening at baseline, 3- and 6-weeks. (n=4 per group per sex) Fold change of **(d)** *Nppb* and ***(e)*** *Nppa* expression from RNA-seq data in WT and CM-Il11-Tg mice 1, 3, and 6 weeks after induction of *Il11* expression (n=4 per timepoint). Statistics: Three-way ANOVA used for echocardiographic data including sex, genotype and time as factors. Differential gene expression was analysed using EdgeR software and corrected using false discovery rate method. Significance is denoted as *p<0.05, **p<0.01, ****p<0.0001

Supplementary Tables

Rosa26-Il11	**Forward**	**GTTTTGGAGGCAGGAAGCACTTGC**
	**Forward**	**GCAGTGAGAAGAGTACCACCATGAGTCC**
	**Reverse**	**CAATGCTCTGTCTAGGGGTTGGATAAGC**
**α-MHC-MerCreMer**	**Forward**	**5'-TCTATTGCACACAGCAATCCA-3'**
	**WT reverse**	**5'-CCAACTCTTGTGAGAGGAGCA-3'**
	**Mutant reverse**	**5'-CCAGCATTGTGAGAACAAGG-3'**

Supplementary Table S1. Primers used for genotyping PCR.

**Target**	**Taqman primer/probe**
Col1a1	Mm00801666_g1
Col3a1	Mm00802300_m1
Fn1	Mm01256744_m1
Il11	Mm00434162_m1
Mmp2	Mm00439498_m1
Mmp9	Mm00442991_m1
Mmp14	Mm00485054_m1
Postn	Mm01284919_m1
Timp1	Mm01341361_m1
Gapdh	Mm99999915_g1

Supplementary Table S2. Taqman probes used for qPCR
